# Impact of Diabetic Retinopathy on Sleep, Mood, and Quality of Life

**DOI:** 10.1167/iovs.18-26108

**Published:** 2019-05

**Authors:** Rupal Morjaria, Iona Alexander, Robert M. J. Purbrick, Rukhsana Safa, Ngaihang Victor Chong, Katharina Wulff, Russell G. Foster, Susan M. Downes, for the SOMNUS Study Group

**Affiliations:** 1 Nuffield Department of Clinical Neurosciences, OMPI, Sir William Dunn School of Pathology, South Parks Road, Oxford, United Kingdom; 2 Oxford Eye Hospital, West Wing, Oxford University Hospitals NHS Trust, Headington, Oxford, United Kingdom; 3 University Hospital Birmingham NHS Trust, Mindelhson Way, Birmingham, United Kingdom; 4 Sussex Eye Hospital, Brighton and Sussex University Hospitals NHS Trust, Eastern Road, Brighton, United Kingdom

**Keywords:** diabetic retinopathy, circadian rhythms, sleep and mood

## Abstract

**Purpose:**

Diabetic retinopathy (DR) is associated with retinal neuronal and vascular damage. DR has previously been shown to affect the photosensitive retinal ganglion cells (pRGCs). PRGCs are essential for the entrainment of circadian rhythms; thus, DR progression could lead to worsening sleep quality and mood. We investigate the relationship between increasing DR severity, and its impact on sleep quality and mood.

**Methods:**

A total of 430 participants with DR, and 303 healthy controls with no ocular disease or preexisting sleep disorders were recruited. DR severity was grouped as follows: 1, mild nonproliferative (NPDR); 2, moderate/severe NPDR; and 3, proliferative diabetic retinopathy (PDR). Sleep, mood, and quality of life were assessed using the Pittsburgh Sleep Quality Index (PSQI), quality of life (SF-36), and Hospital Anxiety and Depression Score (HADS) questionnaires. Data were analyzed by severity of DR, and correlated with sleep, QOL, and mood and compared to controls.

**Results:**

No significant difference between PSQI scores in the DR group or the control group was identified despite severity of DR. Mean anxiety and depression scores were within the normal range for both groups. Despite a lower general health and physical function, the DR group had lower anxiety scores than controls.

**Conclusions:**

These data show that even in severe DR, sleep quality is similar to controls. However, this could be explained by the majority of individuals in this study having good visual acuities in the better eye with a residual population of pRGCs remaining unaffected by DR.

Diabetic retinopathy (DR) is a leading cause of blindness in the working population.^1^,^2^ DR is a chronic progressive condition, which can lead to varying degrees of visual impairment.^3^ Although DR is asymptomatic in the early stages, electrophysiological abnormalities in function of the inner layer of the retina have been recorded as early as 6 months after the diagnosis of diabetes, even in the absence of visible DR. Further electrophysiological changes are seen in longer disease duration.^4^ Animal models of DR show retinal cell dysfunction and morphological and vascular changes including ganglion cell loss (RGC).^5^ One study using optical coherence tomography (OCT) in 126 individuals with DR compared to 227 controls, showed RGC loss with increasing severity of DR leading to progressive visual loss.^6^ Both animal model and human studies have shown that a subpopulation of RGCs, photosensitive retinal ganglion cells (pRGCs) are affected in diabetes^6^–^11^ even though the results are conflicting.^12^,^13^

PRGCs are essential for nonimage forming functions driving circadian biology.^14^ If pRGCs become damaged or destroyed, as would be expected with severe or advanced DR, then functions of circadian biology such as sleep may also be disrupted. DR thus has the potential to affect not only vision but also to disrupt circadian function.

There is evidence to suggest that poor sleep itself contributes to the pathogenesis of DR and development of proliferative DR. Another consideration is that disturbed sleep has been shown to negatively influence immune function and inflammation^15^,^16^ this latter has been implicated in the pathogenesis of DR^17^ and is a target for pharmacological treatments of DR.^18^,^19^ It is therefore difficult to determine whether sleep problems are a consequence of DR, or a risk factor for developing DR, or both are occurring independently of one another.

A meta-analysis and literature review examining the quantity and quality of sleep in diabetes has shown that impairment of either can be a risk factor for developing type 2 diabetes (see Ref. 20 for review) and poor sleep quality and shorter sleep duration have been associated with type 1 diabetes.^21^,^22^ Reduced sleep duration has been proposed as a risk factor for insulin resistance in type 2 diabetes and has been linked to abnormal glucose metabolism and increased diabetes risk.^23^ Not only have deficits in quality and quantity of sleep been shown to predict the onset of diabetes, but are also more prevalent in individuals with diabetes.^20^,^22^,^24^–^28^ However, retinal examination was not reported in any of these studies, so it is not possible to be certain if the reported sleep disturbances are due to pRGC involvement or other factors.

There are four studies investigating sleep quality, specifically sleep duration, in DR^22^,^24^,^27^,^28^; of these, only two report on differing severity of DR on sleep but neither of these studies exclude preexisting sleep conditions such as obesity, restless leg syndrome, pain, nocturia, and obstructive sleep apnea (OSA).^24^,^28^ Thus the observation made by Tan et al.^28^ that their severe group (severe nonproliferative DR, proliferative DR, or clinically significant macular edema) had excessive daytime sleepiness, and a longer than normal sleep time, may be due to the presence of conditions that affect sleep rather than the DR itself.

To our knowledge, this is the first study to examine the impact of varying DR severity on sleep quality, and mood, in a large cohort of individuals with preexisting sleep disorders excluded, compared to healthy controls.

## Methods

A total of 512 Participants with DR (with type 2 diabetes) were recruited from specialist medical retina clinics at the Oxford University Hospitals NHS Trust, Buckinghamshire Healthcare NHS Trust, Royal Berkshire Hospital and Cambridge from 2011 to 2018. We recruited 312 controls over the age of 18 (with no eye disease) between 2015 and 2017, from advertisements in the hospital, press release, staff members, and public information days.

Age, sex, DR grading, any ocular copathology, and visual acuity were recorded. Exclusion criteria included: coexistent eye disease, previous eye surgery, or an intervention that could affect retinal function (unless procedures were for treatment of DR), the presence of any known primary or secondary sleep disorders; past or present alcohol or substance abuse; organic physical or psychiatric conditions; prescription of benzodiazepines; head injury; and a history of continued shift work. Controls were required to have a screening visual acuity of 6/6 or better at recruitment.

A general health questionnaire adapted from the patient health questionnaire was used to confirm eligibility.^29^

This multisite study was approved through the Oxfordshire Research Ethics Committee South Central Oxford B (Ref: 11/SC/0093) and carried out in accordance with the tenets of the Declaration of Helsinki.^30^ Informed consent was obtained from all participants.

### Ophthalmic Examinations

All individuals with known DR underwent ophthalmologic examination including visual acuities, slit lamp examination, dilated fundoscopy, and fundus photography. Diabetic grading was performed using the diabetic grading software tool on the standard EPR system (Medisoft, Sorinnes, Belgium). In most cases, DR was graded in real time in the clinic and the remainder were graded on imaging.

DR was graded according to the international clinical diabetic retinopathy disease severity scale (see Table 1) and participants were classified in to three groups according to the DR grading of the better eye (see Table 2).

**Table 1:** Retinopathy Grades in Individuals With Diabetes

**Clinical Features**	**Description**
No retinopathy	No retinopathy
Microaneurysms only	Moderate DR
More than microaneurysm, but less than severe NPDR	
At least one of: extensive >20 intraretinal hemorrhages in each of the four quadrants, venous beading in 2+ quadrants, IRMA in 1+ quadrant, absence of PDR	Severe NPDR
Neovascularization +/− Vitreous/preretinal hemorrhage	Proliferative DR

**Table 2:** Groups of Participants Recruited

**Groups of Participants Recruited by Severity of DR**	**Numbers**	**Clinical Features**
Healthy controls no diabetes	303	No retinopathy
Mild NPDR	150	Microaneurysms only mild DR
Moderate/severe NPDR	129	At least one of: extensive >20 intraretinal hemorrhages in each of the four quadrants, venous beading in 2+ quadrants, IRMA in 1+ quadrant, absence of PDR
Proliferative DR	151	Neovascularization +/− Vitreous/preretinal hemorrhage

### Assessments of Sleep, Mood, and Quality of Life

Individuals completed self-rated questionnaires assessing sleep, mood, and quality of life (QoL). Subjective sleep quality was assessed using the Pittsburgh Sleep Quality Index (PSQI).^31^,^32^ Mood was assessed using the Hospital Depression and Anxiety Scale (HADS).^33^ The Short Form Health Survey (SF-36)^34^ was used to evaluate QoL over eight domains: physical functioning (PF), role limitation due to physical health (RLPH), role limitation due to emotional problems (RLEP), energy/fatigue (EF), emotional well-being (EMWB), social functioning (SF), pain, and general health (GH). Higher scores represent a more favorable state of health.

The Epworth Sleepiness Scale (ESS)^35^ scores above 10 are considered to indicate excessive daytime sleepiness. BMI was recorded. The season the questionnaires were completed in (season) was also recorded; spring (February to April), summer (May to July), autumn (August to October) and winter (November to January). The morningness-eveningness questionnaire (MEQ)^36^ was used to determine chronotype.

### Statistical Analysis

All statistical analysis was carried out with the assistance of a statistician and analyzed using R-2.15 (R Development Core Team 2012, University of Auckland, Auckland, New Zealand; available at http://www.R-project.org). All data were manually entered into Excel. Nonparametric *T*-tests were used to explore the distribution of age, BMI, chronotype, and quality of life variables between groups. Fisher's exact tests, χ^2^, and Kruskall-Wallis test was used to check for comparability between the DR severity groups.

Data were fitted to a linear regression model. All PSQI and HADS scores were log transformed, and a square root of logMAR taken to meet linearity. A cumulative linked mixed-model (CLMM; ordinal library in R) was used to analyze the PSQI component scores. We present *P* values with significance assumed when *P* < 0.05.

## Results

### DR and Controls Participant Characteristics and Comparisons

A total of 430 individuals with DR were allocated to 3 groups of increasing DR severity. Only 2 participants in the entire DR cohort had >1.00 logMAR in the best eye. A total of 303 controls (9/312 were excluded for incomplete data recording) with excellent visual acuity (logMAR ≤ 0) participated. Table 3 describes the participants demographic characteristics and questionnaire scores.

**Table 3:** Demographics of DR and Control Participants and Individuals With Mild NPDR, Moderate/Severe NPDR, and PDR

	**All DR**	**Controls**	***P*** **Value**	**DR Severity**			***P*** **Value**
			**DR vs. Controls**	**Mild NPDR**	**Moderate/Severe NPDR**	**PDR**	**DR Severity**
*N* (male/female)	430 (199, 231)	303 (83/220)	*P* < 0.0001	150 (72, 77)	129 (63, 66)	151 (64, 87)	NS
Age years, x̄ (SD)	59.61 (13.26)	47.80 (16.30)	*P* < 0.0001	63.11 (12.86)	59.93 (14.45)	56.13 (12.59)	*P* < 0.001
Range	22–92	19–91					
Visual acuity, *n* (%)							
LogMAR, x̄ (SD)	0.079 (0.213)	(303)	N/A	0.07 (0.2)	0.054 (0.2)	0.116 (0.231)	*P* = 0.034
Mild/none (<0.5)	410 (96.7%)	0		143 (97.3%)	125 (96.9%)	140 (95.9%)	
Moderate (>0.5 to <1.00)	12 (2.8%)	0		3 (2%)	3 (2.3%)	5 (3.5%)	
Severe (≥1.00)	2 (0.5%)	0		1 (0.7%)	1 (0.8%)	1 (0.6%)	
Missing	8	0		3	0	5	
BMI (x̄)	28.79 (7.38)	26.47 (6.54)	*P* < 0.0001	28.64 (8.09)	28.23 (7.02)	29.39 (6.99)	*P* = 0.066
Underweight (<18.5)	8 (1.9%)	6 (2.1%)		1 (0.7%)	2 (1.6%)	5 (3.3%)	
Healthy (18.5–24.9)	95 (22.1%)	135 (47.5%)		36 (24.2%)	36 (27.9%)	23 (15.3)	
Overweight (25–29.9)	168 (39%)	89 (31.3%)		65 (43.6%)	45 (34.9%)	58 (38.7%)	
Obese (>30)	159 (37%)	54 (19%)		46 (30.9%)	46 (35.7%)	64 (42.7%)	
N/A	2	0		1 (0.7)	1	0	
PSQI >5, *n* (%)	161 (37.5%)	120 (39.6%)	NS	59 (39.3%)	54 (41.9%)	48 (31.8%)	NS
PSQI >10, *n* (%)	67 (15.51%)	36 (11.88)	NS	25 (20.9%)	23 (17.83%)	19 (12.58%)	NS
PSQI and subscales							
PSQI, x̄ (SD)	5.21 (3.68)	5.45 (3.24)	NS	5.34 (3.79)	5.36 (3.58)	4.97 (3.67)	NS
Quality	0.97 (0.85)	1.05 (0.73)	NS	0.96 (0.98)	1.02 (0.75)	0.92 (0.77)	NS
Latency	0.78 (0.83)	0.79 (0.72)	NS	0.77 (0.83)	0.78 (0.84)	0.79 (0.82)	NS
Duration	0.75 (1.0)	0.69 (0.85)	NS	0.77 (1.01)	0.79 (1)	0.69 (0.97)	NS
Efficiency	0.87 (1.14)	0.71 (0.97)	NS	0.95 (1.2)	0.88 (1.12)	0.81 (1.09)	NS
Disturbance	1.24 (0.53)	1.29 (0.56)	*P* = 0.0167	1.25 (0.55)	1.24 (0.48)	1.23 (0.55)	NS
Medication	0.10 (0.47)	0.16 (0.58)	NS	0.11 (0.51)	0.1 (0.5)	0.09 (0.44)	NS
Daytime dysfunction	0.24 (0.83)	0.75 (0.76)	*P* = 0.0044	0.61 (0.84)	0.55 (0.8)	0.44 (0.81)	*P* = 0.004
Chronotype, x̄ (SD)	61.31 (10.09)	59.27 (9.58)	*P* = 0.008	62.44 (10.53)	61.04 (10.39)	59.98 (10.28)	*P* = 0.052
Anxiety, x̄ (SD)	3.64 (3.10)	5.61 (3.61)	*P* < 0.0001	3.50 (3.93)	3.94 (3.28)	3.53 (2.97)	*P* = 0.0078
Normal range, *n* (%)	383 (88.65%)	221 (72.9%)		136 (90.7%)	110 (85.3%)	134 (88.7%)	
Depression, x̄ (SD)	2.71 (2.63)	2.88 (2.93)	NS	2.65 (2.38)	2.43 (2.61)	3.01 (2.86)	*P* = 0.0104
Normal range, *n* (%)	408 (94.4%)	277 (91.4%)	NS	144 (96.7%)	122 (94.6%)	139 (92.1%)	
ESS, x̄ (SD)	6.91 (3.97)	6.20 (4.22)		7.18 (3.96)	6.66 (4.07)	6.85 (3.92)	
Normal range, *n* (%)	324 (75%)	235 (77.6%)		112 (74.7%)	108 (83.7%)	123 (81.5%)	
Quality of life, x̄ (SD)							
Physical functioning	75.60 (26.83)	86.53 (20.24)	*P* < 0.0001	77.93 (24.2)	77.21 (25.02)	72.12 (30.36)	NS
Physical limitations	77.24 (36.84)	89.27 (36.87)	*P* < 0.0001	81.18 (32.61)	79.65 (34.63)	71.36 (41.87)	NS
Emotional limitation	92.62 (22.09)	90.54 (25.29)	NS	92.12 (21.78)	93.49 (20.52)	92.27 (23.86)	NS
Energy/fatigue	61.82 (18.81)	62.62 (19.44)	NS	64.53 (17.68)	60.19 (19.23)	60.66 (19.33)	NS
Emotional wellbeing	82.81 (14.27)	76.16 (16.67)	*P* < 0.0001	84.42 (13.42)	81.49 (14.48)	82.25 (14.89)	NS
Pain	79.80 (23.77)	81.72 (21.85)	NS	88.3 (21.25)	88.48 (21.74)	83.42 (25.15)	NS
Social functioning	86.7 (22.87)	88.20 (19.89)	NS	81.02 (22.13)	79.57 (25.88)	78.87 (23.69)	NS
General health	56.53 (22.84)	71.77 (19.41)	*P* < 0.0001	60.25 (21.70)	56.9 (22.71)	52.57 (23.66)	*P* = 0.046

x̄, mean; NS, not significant; SD, standard deviation.

Individuals with DR were older than controls (*W* = 37,546, *P* < 0.0001) with more females in the control group (Fisher's exact test, *P* < 0.0001). Chronotype (*W* = 58,025.5, *P* = 0.008) and BMI (*W* = 41261, *P* < 0.0001) were not comparable between DR and controls. The seasons were not comparable within either group (DR: *χ*^2^[3] = 9.98, *P* = 0.001872; nor controls: (*χ*^2^[3] = 18.149, *P* = 0.0004099).

Poor sleep (PSQI score >5) was reported in <40% of all participants and <25% of participants reported poor ESS. A total of 11.35% of individuals with DR and 27.1% of controls reported a possible anxiety disorder. A total of 5.6% of individuals with DR and 8.6% of controls reported a possible depressive disorder.

QoL scores were worse for individuals with DR in the following domains; PF (*W* = 81826, *P* < 0.0001), RLPH (*W* = 74875, *P* < 0.0001), and GH (*W* = 898456, *P* < 0.0001). DR had better EMWB scores (*W* = 48200, *P* < 0.0001).

### DR Sleep Quality, Daytime Sleepiness, Mood, and Quality of Life Compared to Controls

Individuals were equally likely to experience poor sleep quality (PSQI score >5; glm [family = binomial], *P* = 0.231793, McFadden's *R*^2^ = 0.0188). An increased BMI was associated with poor sleep (*P* = 0.000957) and all other predictors (age, sex, and chronotype) were not significant (*P* > 0.5).

Overall sleep quality scores were comparable (*P* = 0.2759, *R*^2^ = 0.1667). Age (*P* = 0.765), sex (*P* = 0.2578), chronotype (*P* = 0.310), season (*P* > 0.05), daytime sleepiness (*P* = 0.180), depression (0.1798), EMWB (0.213), and GH (*P* = 0.5623) had no impact on sleep scores. Sleep quality worsened as BMI increased (*P* = 0.0046), anxiety worsened (*P* = 0.000464), and as RLPH (*P* = 0.004911) worsened. There was a trend for sleep quality to worsen with worsening PF (*P* = 0.0696).

### Sleep Components Underpinning Sleep Quality Score Controls Versus DR

Individuals with DR had less sleep disturbance (*P* = 0.0167) and better daytime functioning (*P* = 0.00438) than controls. All other components were comparable (see Table 3).

### Mood Controls Versus DR

Individuals with DR were less anxious than controls (*P* < 0.0001, *R*^2^ = 0.5111). Neither sex (*P* = 0.3869), chronotype (*P* = 0.88), season (*P* > 0.05), PF (*P* = 0.215), nor RLPH (*P* = 0.1306) had any impact on anxiety. Increased age was associated with worsening anxiety (*P* = 0.000245). As anxiety worsened, so did sleep quality (*P* = 0.000464), depression (*P* < 0.0001), GH (*P* = 0.0136), and EMWB (*P* < 0.0001). There was a trend for increased anxiety as BMI increased (*P* = 0.063).

Depression scores were comparable between groups (*P* = 0.214674, *R*^2^ = 0.463). Sex (*P* = 0.724), chronotype (*P* = 0.161), BMI (*P* = 0.1669), season (*P* > 0.05), daytime sleepiness (*P* = 0.33533), sleep quality (*P* = 0.1798), and RLPH (*P* = 0.102) had no impact. Older individuals were more depressed (*P* = 0.0001). Higher anxiety scores (*P* < 0.0001), worse PF (*P* = 0.00022), GH (*P* < 0.0001) and EMWB (*P* < 0.0001) were associated with worsening depression.

### Mood Controls Versus DR Severity

Individuals with PDR had better sleep scores (*P* = 0.041) than controls (*P* > 0.05, *R*^2^ = 0.1665), all other comparisons were not significant (*P* > 0.05). Anxiety scores were lower in all the DR, irrespective of severity than controls (*P* < 0.001, *R*^2^ = 0.432). Individuals with moderate/severe NPDR were less depressed than controls (*P* < 0.003. *R*^2^ = 0.4447) all other comparisons were not significant (*P* > 0.05; see Figure).

**Figure:**
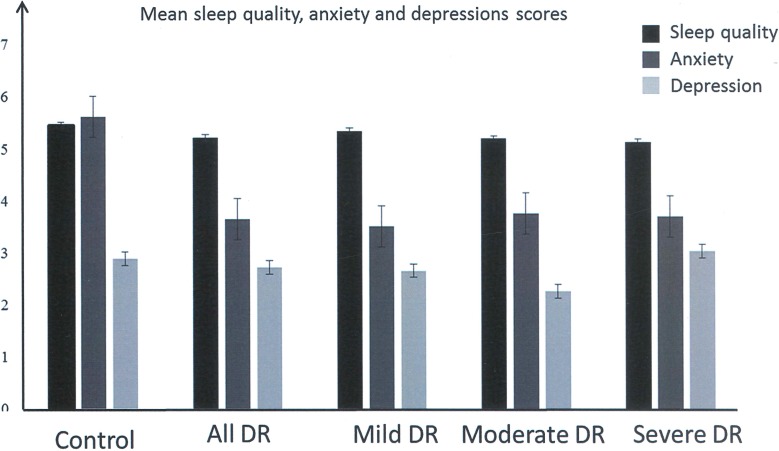
Sleep quality, anxiety, and depression scores in control participants and individuals with DR and the different DR severity.

### Participants Characteristics According to DR Severity Alone

Age was not comparable between groups (*χ*^2^ = 25.68, *P* = < 0.0001); individuals with mild NPDR were older than those with moderate/severe NPDR (*P* = 0.022) and PDR (*P* = < 0.0001). All other comparisons were not significant (*P* > 0.05).

Chronotype scores were not comparable between DR groups (*χ*^2^ = 5.911[2], *P* = 0.052); individuals with mild DR were later chronotypes than those with PDR (*P* = 0.048) but moderate/severe NPDR and PDR were similar (*P* > 0.05). BMI were comparable between groups (χ^2^ = 5.4466[2], *P* = 0.06566). Sex (*χ*^2^ = 37.05[2], *P* = 0.4703), season (*χ*^2^ = 3.94[6], *P* = 0.6837), were equally balanced groups.

Poor sleep (PSQI score >5) was reported in 42% of individuals with mild NPDR and 40% with moderate/severe NPDR, and 32% of those with PDR. Over 74% of individuals with DR reported ESS scores in the normal range (ESS <10). Over 85% of individuals reported anxiety scores in the normal range (HADS-A ≤ 7) and over 90% reported depression scores in the normal range (HADS-D ≤ 7).

QoL scores were comparable between DR severities except GH (*χ*^2^ = 6.152, *P* = 0.046); mild NPDR had better GH scores than those with PDR (*P* = 0.046), but moderate/severe NPDR and PDR GH scores were similar (*P* > 0.05; Bonferroni correction applied).

### Visual Acuity and Sleep Quality

Visual acuity scores were not comparable between groups (χ^2^ = 6.794[2], *P* = 0.034); PDR had significantly worse VA than moderate/severe NPDR (*P* = 0.043). There was no relationship between visual acuity (logMAR) and sleep quality (*P* = 0.327, *R*^2^ = −0.002). However, only two participants were severely sight impaired (defined as LogMAR VA >1.0).

### DR Severity and Sleep Quality, Daytime Sleepiness, Mood, and Quality of Life

Increasing DR severity was not associated with poor sleep quality (PSQI score >5; glm [family = binomial], *P* > 0.05, McFadden's *R*^2^ = 0.156; see Figure).

Overall sleep scores were comparable between increasing DR severity (*P* > 0.05; *R*^2^ = 0.1). As BMI increased (*P* = 0.0031), depression scores worsened (*P* = 0.007) so did sleep quality. Age (*P* = 0.863), sex (*P* = 0.679), chronotype (*P* = 0.683), season (*P* >0.05), ESS (*P* = 0.168), anxiety (*P* = 0.469) had no impact on sleep quality.

Individuals with PDR were less anxious than those with moderate/severe NPDR (*P* = 0.0078) and a trend for worse scores than those with mild PDR (*P* = 0.0866). Anxiety scores were comparable between mild and moderate/severe NPDR (*P* = 0.3307). Individuals with PDR had worse depression scores than those with moderate/severe NPDR (*P* = 0.0104), but not those with mild NPDR (*P* = 0.1381). Scores were comparable between for those with mild NPDR and moderate/severe NPDR (*P* = 0.1381; see Figure).

### Sleep Components Underpinning Sleep Quality Score

Individuals with PDR had better DF than those with mild NPDR (*P* = 0.004) but not those with moderate/severe NPDR (*P* = 0.06). DF scores were comparable between individuals with mild and moderate/severe NPDR. Sleep quality, sleep latency, sleep duration, sleep disturbance, and use of sleep medications were similar between severity groups (*P* > 0.05).

## Discussion

This is the first study to investigate the impact of DR, and DR of varying severity on sleep, mood, and quality of life in a large cohort of patients without preexisting sleep disorders, compared to controls. There is very little in the literature regarding the impact of DR on sleep, and none of the four studies^22^,^24^,^27^,^28^ that report on this have investigated whether individuals with DR have worse sleep than controls, or whether there is a relationship between DR severity and sleep. Ours is also the first study to investigate sleep quality, having excluded confounding factors.

The finding in this study that DR severity did not have an impact on sleep quality was unexpected; however, there were only two individuals with severe visual impairment. This finding was similar to the study by Jee et al.^24^ where they showed no significant association between their severe DR group (85 with severe nonproliferative, proliferative, and macular edema), and sleep duration.

Tan et al.^28^ investigated sleep duration and daytime sleepiness in 1231 individuals with diabetes (129 with DR and 77 with severe DR [severe nonproliferative, proliferative, and macular edema). Like Jee et al.,^24^ they report an association between DR and sleep duration, but only report an incidence of long sleep in severe DR and increased daytime sleepiness. However, no comparisons were made between their severe DR, DR, and no DR, making it impossible to determine whether increasing severity of DR is associated with worsening sleep quality. In our study, there was no difference in sleep duration between individuals with DR and controls, nor with increasing DR severity. This suggests that long and short sleep may be due to factors other than RGC integrity or circadian dysfunction. Our results also support those of Meng et al.^27^ who reported no increased incidence of increased sleep latency in individuals with DR although their sample size was small, and there was no record of DR.

Despite the potentially confounding variable where individuals with DR were older than controls, as well as the sex difference,^37^ age had no impact on sleep quality and sleep scores were comparable. Although a sex difference was reported, this may be underpinned by the control group (which had more females) as there was no impact of sex on sleep quality in DR. There was also no impact of season on sleep quality or mood, which may reflect a misbalance of season distribution within groups. The BMIs recorded in our DR cohort were higher than in the control group despite excluding any individuals reporting sleep apnea. Although Bjorvatn^21^ reported an increased BMI may be indicative of sleep problems in the absence of sleep apnea, we found no increased prevalence of poor sleep among the DR group compared to controls. However, we did exclude individuals with preexisting sleep disorders, which may reflect the difference in the findings. Lamond et al.^23^ proposed that impaired sleep quality in diabetes was associated with complications in diabetes (e.g., pain at night), and it may be that, rather than impaired pRGC function that underpins poor sleep in diabetes. However, in our cohort, not only was there no difference in sleep disruption, there was also no difference in pain scores between DR and control groups nor between increasing DR severity groups. This finding also suggests that our cohort did not include individuals with advanced stages of diabetes such as polyneuropathy, although this has not been confirmed.

Although all domains of general health, as defined by the SF-36, were comparable between individuals with increasing severities of DR, controls had better physical health and general health. However, these were not significant enough to have an impact on sleep quality suggesting QoL did not impact perceived sleep quality. We also found that individuals with DR, although were less well in terms of general health and quality of life, were significantly less anxious than controls, but were equally likely to be depressed.

We deliberately restricted our study to those who did not have a known sleep or mood disorder. Although two groups reported poor sleep in a significant number of participants with diabetes (71%^25^ and 55%^38^) they did not exclude previous sleep or mood disorders or medications that may affect sleep quality. This may explain why their reported SCRD is substantially higher than we found in our study, wherein only 37.5% had sleep disruption and only 42.4% of our controls reported poor sleep. It is possible that our strict exclusion criteria, (a history of preexisting sleep disorders, sleep medications, and mood disorders) may have led to a sample bias of a particularly resilient group of individuals with DR. However, these exclusion criteria were essential to disentangle the potential impact of DR assumed to be related to pRGCs loss, rather than due to factors unrelated to pRGC function. We also did not have any severely affected patients, which could be consequent upon effective screening with early and improved treatments for DR.^39^ Despite having a group of individuals classified as having severe DR, only two in this group were registered as severely sight impaired, thus explaining the absence of a significant relationship between visual acuity and sleep quality.

Another way of assessing pRGC function is pupillary responses; Feigl et al.^8^ identified a trend for impaired pRGC function in DR in their data. We found no evidence to suggest that increasing severity of DR in our cohort led to impaired sleep quality or mood, and presume that there are remaining functioning pRGCs. This would be expected based on the visual acuities of the majority of our DR participants. Although pupillometry is speculatively used to infer pRGC integrity, retinal nerve fiber layer thickness,^40^ and pRGC function,^41^ neither pupillometry nor optical coherence tomography were assessed in our study. Although we may have expected a reduction in pRGC integrity, pRGC functionality (as determined by sleep quality) is sustained. The number of pRGCs required to sustain circadian biology is not known, but in this visually intact population, where comorbidities are accounted for, circadian biology appears to be intact. Future studies comparing individuals with PDR including those with severe visual impairments should consider functional data. However, there is currently no validated reliable measure of pRGC function in humans.

However, we would have expected to see some abnormalities in sleep in our cohort even in the presence of preserved visual acuities as there is evidence to suggest that diabetes leads to morphological changes in pRGCs, rather than actual cell death, which leads to changes in circadian biology, including a delay in entrainment.^10^ It is usually the case that the longer diabetes has been present, that the risk of developing DR increases. We do not have information regarding the duration of diabetes for our cohort and this may be the reason that we have not identified sleep disturbance. It might be predicted that our severe group would have had diabetes for longer than the other groups; therefore, this group might have been expected to demonstrate sleep wake disturbance. However, we only investigated self-reported sleep disturbance and not entrainment. In our cohort, sleep duration and sleep latency (delay to sleep) were not different between any of the groups. Short and long sleep, and worse sleep quality are related to increased HbA1c levels,^42^ and increased blood glucose levels are related to increased incidence of DR and of DR severity.^43^ Although we did not measure HbA1c, future studies should consider it as our strict exclusion criteria may have biased our sample individuals with DR and good diabetic adjustment. Although it is possible that objective measures of sleep latency and sleep duration used in this study are less sensitive than the behavioral measures under environmental control used by Lahouaoui et al.,^10^ it is unlikely that DR leads to circadian disruption in the absence of severe sight impairment. Thus, in this group, it appears that pRGC function is not impaired in DR with preserved vision.

In conclusion, these results suggest that in a population with good visual acuity, DR has no negative impact on sleep quality or mood. It is possible that pRGCs function has not been sufficiently compromised to have an impact on subjective sleep measures. For those who have more significant loss of vision, and by implication retinal tissue loss, this may not be the case, and their sleep may be disrupted. Further studies in this specific population would be required to confirm this. However, these findings in this cohort of patients with varying severity of DR attending three different diabetic clinics is encouraging news for an aging population with an increasing prevalence of diabetes.

## Appendix

### SOMNUS Group

Oxford University Hospitals NHS Trust and Nuffield Laboratory of Ophthalmology, University of Oxford: Susan Downes (PI), Rukhsana Safa, Katharina Wulff, Iona Alexander, Sophie Marlowe, Colm Andrews, Caroline Justice, Alexina Fantato, Russell Foster.

Buckinghamshire Healthcare NHS Trust: Hiten Sheth (PI), Judith Abrams, and Katarina Manso.

Hinchinbrook Hospital: Rupert Bourne (PI), Paula Turnbull.

Moorfields Eye Hospital: Anthony Moore and Phil Hykin (PI), Emily Summers.

Royal Berkshire NHS Foundation Trust: Muhammed Tahir (PI), Sue Nuth, and Emma Craig.

Bristol Eye Hospital: Amanda Churchill (PI), Eleanor Hiscott.

Central Manchester University Hospitals NHS Foundation Trust: Graeme Black (PI).

Portsmouth Hospitals NHS Trust: James Kirwan (PI), Mini David.

University Hospital Wales: Marcela Votruba (PI).

Frimley Health NHS Foundation Trust: Geeta Menon (PI), Ganga Pathinayake, and Nora Mistersky.
